# Supplementary material for: MetaRibo-Seq measures translation in microbiomes
Source: Nat Commun. 2020 Jun 29;11:3268. doi: 10.1038/s41467-020-17081-z (PMC7324362; doi:10.1038/s41467-020-17081-z)
Supplement: Supplementary file 10 — Supplementary Data 7 [file 41467_2020_17081_MOESM10_ESM.zip › File2/Confidence_High_Taxonomy/378103_out.krona.html]

Javascript must be enabled to view this page.

members
magnitude
magnitudeUnassigned
count
unassigned
taxon
rank

378103\_out

21

superkingdom
2
21

phylum
976
21

class
200643
21

order
171549
21

21
family
815

21
genus
816

818
species

SRS013215\_contig\_number\_2120
1

species
246787

SRS013800\_contig\_number\_13613
1

1

SRS064276\_contig\_number\_16276
2292935
species

387090
species

SRS050299\_contig\_number\_contig-100\_872.82518
1

8

SRS013951\_contig\_number\_21393SRS015579\_contig\_number\_22402SRS020233\_contig\_number\_52925SRS020394\_contig\_number\_15801SRS023583\_contig\_number\_16834SRS023715\_contig\_number\_2776SRS024549\_contig\_number\_4098SRS042628\_contig\_number\_13047
species
821


SRS013940\_contig\_number\_5156SRS057717\_contig\_number\_1388SRS075341\_contig\_number\_615SRS077392\_contig\_number\_43926SRS101268\_contig\_number\_263SRS1041038\_contig\_number\_14682SRS140645\_contig\_number\_999
species
357276
7

1

SRS065504\_contig\_number\_15771
species
47678


SRS023346\_contig\_number\_7261
species
817
1
